# Pulmonary metastatic paraganglioma with angiosarcomatous differentiation: a rare case report and literature review

**DOI:** 10.11604/pamj.2025.51.88.48376

**Published:** 2025-08-07

**Authors:** Shengmin Huang, Jin Zheng, Omid Haravi, Richard Wu, Jen-Chin Wang

**Affiliations:** 1 Department of Internal Medicine, Brookdale University Hospital Medical Center, Brooklyn, New York, United States of America,; 2 Division of Hematology and Oncology, Brookdale University Hospital Medical Center, Brooklyn, New York, United States of America,; 3 Department of Pathology, Brookdale University Hospital Medical Center, Brooklyn, New York, United States of America

**Keywords:** Paraganglioma, transformation tumors, lung, radiation-associated angiosarcoma, case report

## Abstract

Paragangliomas are rare neuroendocrine tumors that can rarely undergo malignant transformation. Angiosarcomatous differentiation is an exceptionally uncommon event with limited reports. This case describes a patient with a previously irradiated vagal paraganglioma that transformed into a metastatic pulmonary paraganglioma with angiosarcomatous features, highlighting the importance of long-term surveillance in irradiated paragangliomas. The patient presented with shortness of breath due to persistent pleural effusion. Despite receiving thoracentesis and palliative radiation therapy, the patient ultimately expired from hypoxic respiratory failure after two months. We add to the limited literature on sarcomatous transformation in paragangliomas and emphasize the importance of considering secondary malignancies in paragangliomas with unusual transformation, and potentially worse prognosis.

## Introduction

Paragangliomas (PGLs) are tumors arising from extra-adrenal chromaffin cells, typically located along the sympathetic or parasympathetic chains, and are seldom found in the mediastinum or thoracic cavity. Angiosarcoma is a rare, highly aggressive malignancy originating from endothelial cells of blood or lymphatic vessels, most commonly affecting the skin of the head and neck. While sarcomatous transformation of PGLs has been reported in only two cases in the literature, no prior cases of angiosarcomatous differentiation have been documented. Notably, both previously reported cases involved prior radiation therapy, raising concern for radiation-associated malignant transformation. Here, we present a case of metastatic paragangliomas with angiosarcomatous differentiation, presenting as multiple pulmonary nodules in a patient who had undergone radiation therapy for a carotid body paraganglioma four years earlier.

## Patient and observation

**Figure 1:**
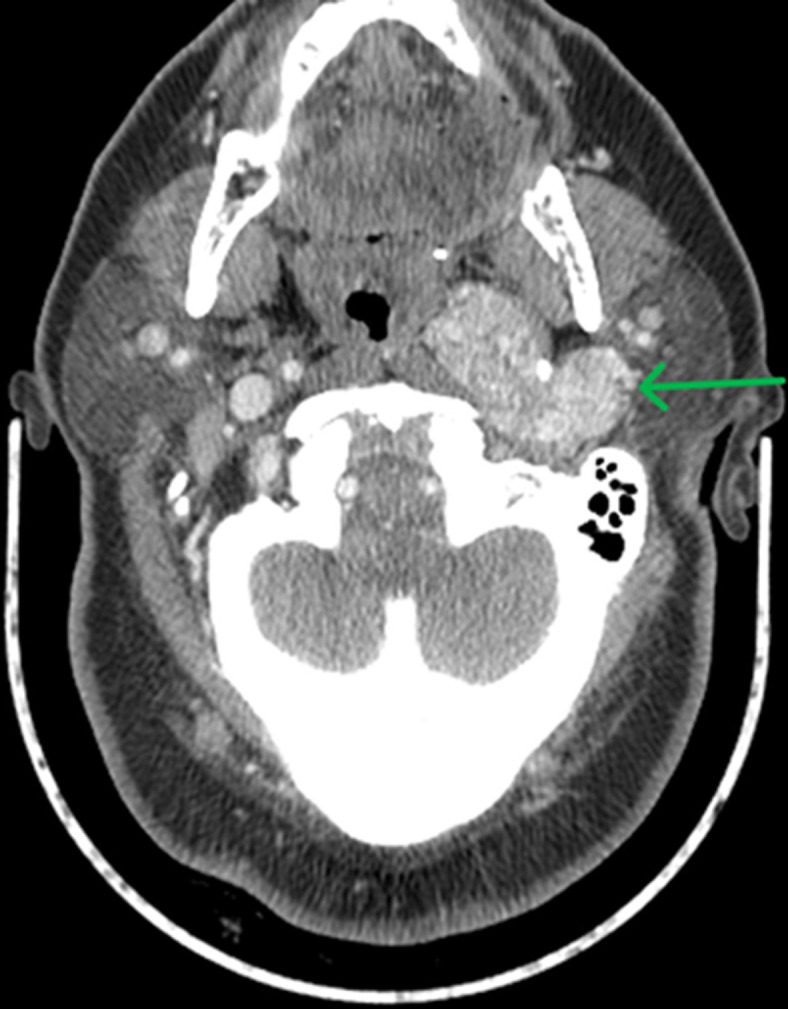
axial CT image of the skull base demonstrating a 6.2 cm hypervascular mass (green arrow) located at the left carotid bifurcation, extending superiorly into and above the left jugular foramen

**Patient information:** a 79-year-old female presented to the Brookdale Hospital Emergency Room with progressive dyspnea over one month, accompanied by a productive cough with blood-tinged sputum and an unintentional weight loss of 15 pounds in 2024. Her medical history was significant for chronic bilateral lymphedema and vaginal intraepithelial neoplasia (VAIN). It is worth noting that she had a vagal paraganglioma of the left carotid artery diagnosed in 2020, initially presenting with persistent hoarseness and inner ear pain. A computed tomography (CT) scan at that time revealed a 6.2 cm solid, hypervascular mass on the left side of the neck, located at the bifurcation of the left carotid artery and extending superiorly into and above the left jugular foramen (Figure 1). Positron Emission Tomography (PET)/Computed Tomography (CT) demonstrated an avid 8 x 4 cm mass with extension into multiple critical regions, including the internal jugular vein, sigmoid sinus, left jugular foramen, adjacent occipital skull base, hypoglossal canal, deep parotid space, and oropharyngeal parapharyngeal space. The patient subsequently underwent external beam radiation therapy, receiving 180 cGy in 25 fractions over the course of five weekly sessions. The lesion showed gradual improvement.

**Clinical findings:** on physical examination, the patient appeared in moderate distress due to shortness of breath. Auscultation revealed decreased breath sounds on the right side, dullness to percussion, and no wheezing or stridor. Bilateral lower extremity non-pitting edema was also noted. Lab results showed elevated normetanephrine of 0.96 nmol/L (normal finding is less than 0.9 nmol/L) with decreased eGFR.

**Timeline of current episode:** in March 2024, a CT scan was performed, revealing multiple ill-defined nodules in the left lung. A few days later, biopsies of two masses were carried out.

**Figure 2:**
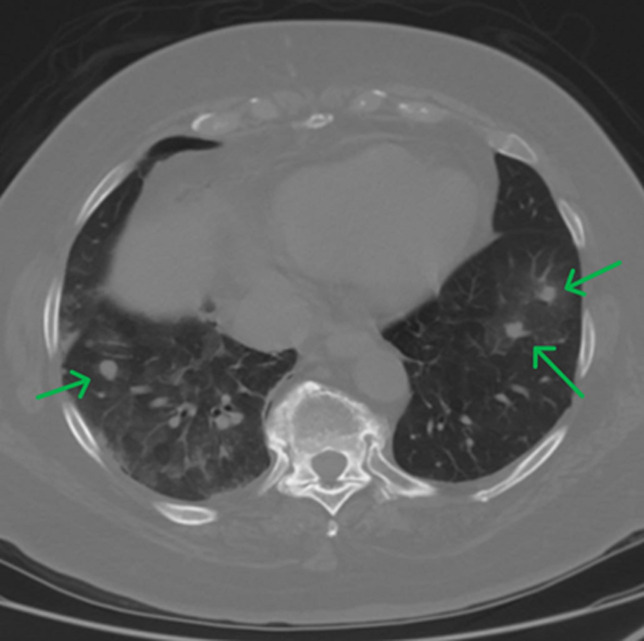
chest CT angiogram with intravenous contrast revealing bilateral scattered pulmonary nodules as well as moderate-sized right pleural-based masses, which are compatible with metastatic disease (green arrow)

**Figure 3:**
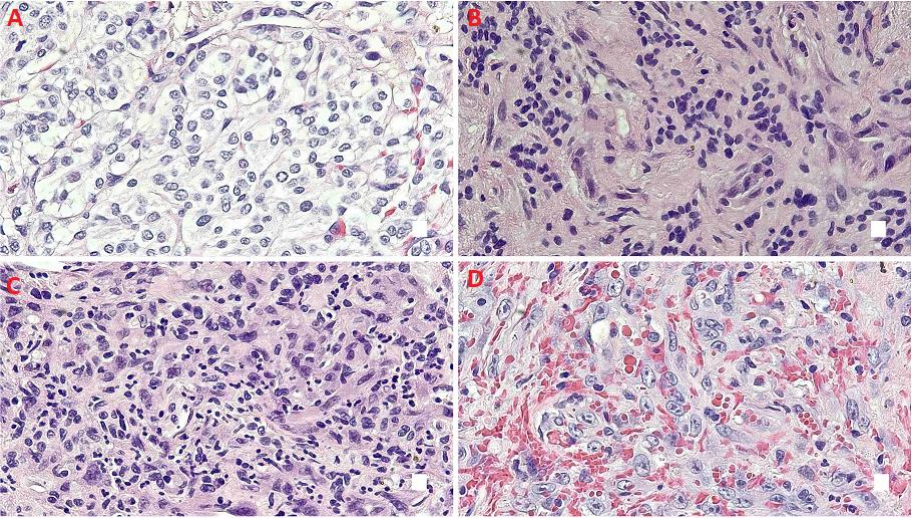
pulmonary metastatic malignant paraganglioma with angiosarcomatous differentiation, metastatic lesion exhibits heterogeneous histologic features: A) typical paraganglioma morphology; B) areas of atypical paraganglioma characterized by a high nuclear-to-cytoplasmic (N/C) ratio; C) regions showing malignant sarcomatous transformation with anaplastic features; D) foci demonstrating angiosarcomatous differentiation

**Diagnostic assessment:** initial chest CT revealed multiple ill-defined nodules in the left lung and a large right-sided pleural effusion causing compressive atelectasis. Subsequent CT imaging without contrast confirmed bilateral pulmonary nodules and moderate-sized pleural-based masses (Figure 2). An IR-guided lung biopsy of the right upper lobe pleural-based nodule was conducted, which was inconclusive, and a video-assisted thoracic surgery biopsy showed two nodules: one measuring 0.8 cm and the other 0.4 cm in size. The larger nodule present in the right lower lobe wedge resection consists of two populations of neoplastic cells. One population consists of well-differentiated round epithelioid cells, with small, round nuclei, present in nests, which are paragangliomas. The second population consists of malignant epithelioid cells with a moderate amount of cytoplasm, nuclei with prominent nucleoli and quite irregular nuclear membranes, indicating a malignant component of angiosarcomatous differentiation (Figure 3).

**Figure 4:**
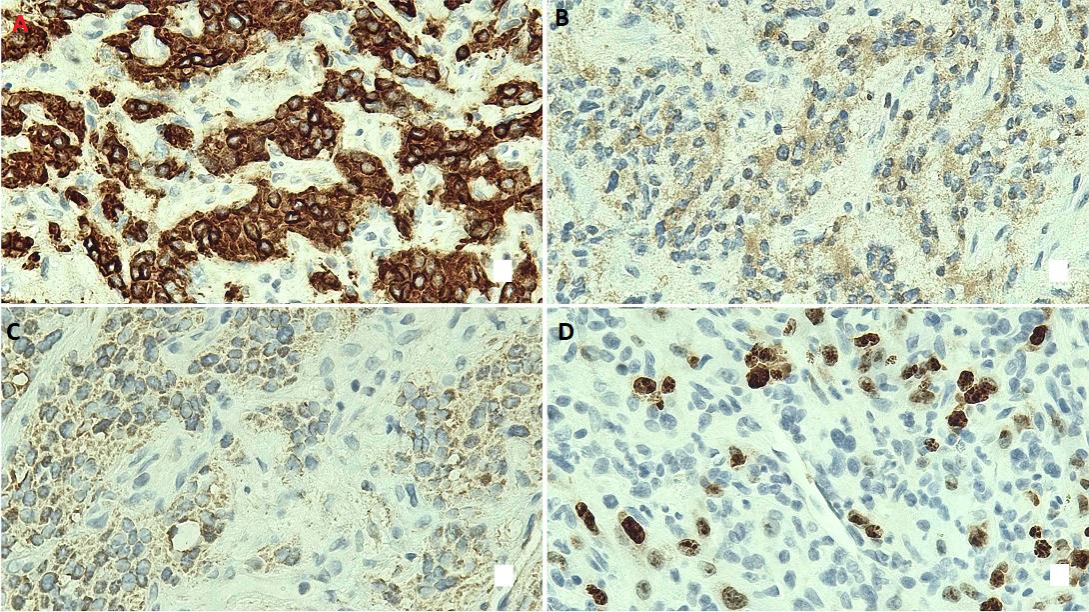
immunohistochemical profile of pulmonary metastatic malignant paraganglioma with angiosarcomatous changes, the metastatic malignant paraganglioma demonstrates diffuse positivity for CD56 (A), chromogranin (B), and synaptophysin (C); the tumor cells are negative for HHV8, SOX-10, TTF-1, S-100, GATA-3, p40, CK5/6, and napsin A; the angiosarcomatous component shows strong positivity for CD31, ERG, and CK8/CK18; retained expression of SDHB and SMARCA4 is observed, the proliferative index (Ki-67) of the neoplastic cells is approximately 20-30% (D)

Immunohistochemical studies showed that both tumor populations were positive for vimentin. The large atypical cells expressed CD56, chromogranin, synaptophysin, GATA-3, and INSM-1, while negative for TTF-1, PR, CD34, CK5/6, napsin A, CK7, AE1/AE3, CK OSCAR, CK8/18, CK CAM5.2, p40, HHV-8, and SOX-10. The small round cells were positive for CK-CAM5.2, cytokeratin 8/18, ERG, and CD31, and negative for HHV-8, SOX-10, TTF-1, S-100, GATA-3, p40, CK5/6, napsin A, with retained SDHB and SMARCA4 expression. Ki-67 proliferation index was approximately 20-30%. Genomic profiling revealed a low TMB (2.3 mut/Mb), microsatellite stability, and PD-L1 negative (Figure 4).

**Diagnosis:** the diagnosis of paraganglioma with angiosarcomatous differentiation was made, based on the histological and immunohistochemical features.

**Therapeutic interventions:** the patient underwent thoracentesis for symptom relief.

**Figure 5:**
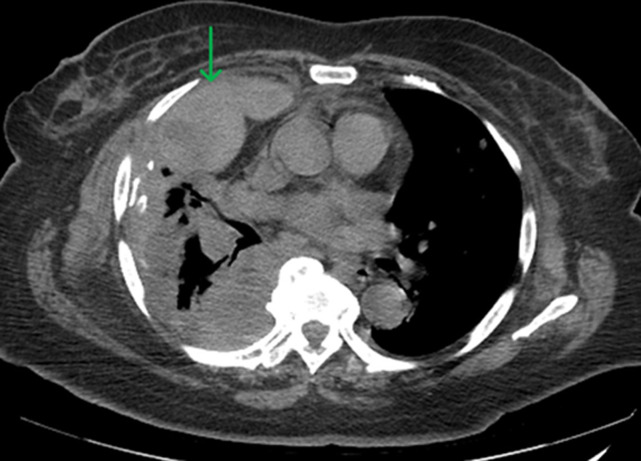
contrast-enhanced chest CT angiogram performed for acute respiratory exacerbation, revealing large multiloculated right pleural effusion with lobar atelectasis and right hemothorax volume loss, variable sized nodules in the left lung suspicious for metastasis and suspected right upper lobe “pneumonectomy” like changes with adjacent solid appearing 10 cm bilobed lesion concerning for a pulmonary malignancy (green arrow)

**Follow-up and outcome of interventions:** the patient was readmitted one month later with worsening shortness of breath, attributed to recurrent right pleural effusion with adjacent atelectasis and/or consolidation, likely secondary to pneumonia. Chest CT revealed a large multiloculated right pleural effusion with lobar atelectasis, right hemothorax with volume loss, and variably sized nodules in the left lung concerning for metastasis. Additionally, there was marked right upper lobe volume loss and architectural distortion, along with a solid-appearing, 10 cm bilobed lesion suspected of pulmonary malignancy (Figure 5). As chest tube drainage provided only partial symptomatic relief, the patient was recommended for palliative radiation therapy with 30 Gy in 10 fractions over two weeks. Despite these interventions, the patient ultimately expired from hypoxic respiratory failure.

**Patient perspective:** the patient initially accepted radiation therapy for her vagal paraganglioma, hopeful it would control the disease. She remained optimistic during follow-up as the tumor showed gradual improvement. Unfortunately, the disease recurred a few years later with more severe symptoms and rapid progression. Despite appropriate treatment, her condition deteriorated quickly, and she ultimately expired. The perspective is based on our observations during her care; throughout her treatments, she appeared to face her illness with strength and expressed appreciation for the medical support she received.

**Informed consent:** it was obtained from the patient´s family member.

## Discussion

Paragangliomas (PGLs) are rare neuroendocrine tumors arising from extra-adrenal chromaffin cells, originating from paraganglia distributed along supradiaphragmatic (parasympathetic) nerves in the head, neck, and mediastinum; the pre- and paravertebral sympathetic chains; or sympathetic nerve fibers innervating the pelvic and retroperitoneal organs. Although these tumors predominantly occur in well-defined anatomic locations, cases of PGLs arising outside the usual distribution of sympathetic and parasympathetic paraganglia have been reported, further expanding the spectrum of these neoplasms.

PPGLs stand for pheochromocytoma and paraganglioma. The primary difference between paraganglioma and pheochromocytoma (PCCs) is their location; PCCs form in the adrenal gland, and PGLs form outside the adrenal gland. The prevalence of PPGLs ranges from 0.1% to 0.6%. Of these, 80-85% of cases are PCCs, while 15-20% are PGLs [1]. Paragangliomas, like pheochromocytomas, are neuroendocrine tumors producing catecholamines. Pheochromocytomas produce both adrenaline and noradrenaline, while paragangliomas only secrete noradrenaline due to the lack of phenylethanolamine N-methyltransferase (PNMT). Excess catecholamines trigger symptoms such as episodic hypertension, tachycardia, diaphoresis, headaches, and palpitations. Some paragangliomas are biochemically silent and incidentally found on imaging.

Paragangliomas (PGLs) predominantly arise from infradiaphragmatic sympathetic ganglia, with only about 1-2% occurring in the thoracic region. Metastatic paragangliomas are more commonly found in the lungs than primary pulmonary paragangliomas [2]. Given the patient´s prior history of carotid body paraganglioma and the Ki-67 index was approximately 30%, these findings are highly suggestive of a malignant paraganglioma and an increased potential for metastasis. In this case, the presentation is more consistent with metastatic rather than primary pulmonary paraganglioma.

An extremely rare phenomenon involves the presence of two distinct tumor types within a single nodule, where a paraganglioma may either coexist with other neural crest-derived tumors or undergo transformation into a different tumor type. Transformation usually occurs into sarcoma, especially malignant peripheral nerve sheath tumors (MPNSTs). A review of the literature identified only two previously reported cases, as summarized in Table 1 [3,4]. In both cases, the biopsies revealed sarcomatous differentiation. Both patients are female and showed positive for S100, with one patient having a history of multiple paraganglioma syndrome. However, to our knowledge, no previously reported case has demonstrated malignant angiosarcomatous differentiation, making our case a novel presentation. The tumor in our patient lacked SOX10 and S100 expression, further distinguishing it from previously reported cases and suggesting an alternative oncogenic pathway. The patient we presented showed two distinct tumor nodules that were identified: one exhibiting classic histopathologic features of a paraganglioma, and the other displaying atypical paraganglioma cells with sarcomatous and angiosarcomatous differentiation. Based on the immunoprofile and pathological findings, it was suggested that the angiosarcomatous differentiation is more likely a malignant transformation arising from the pre-existing paraganglioma. A case presented by Razack *et al*. [4] in which a 44-year-old female with a cervical small cell variant of paraganglioma exhibited sarcomatous transformation. In that case, the tumor consisted of both paraganglioma and sarcoma components, which eventually transformed into S100 and SOX10-positive sarcoma resembling an MPNST following chemoradiation therapy. It is worth noting that sarcomatous differentiation was observed 16 months after radiation therapy. Although the diagnostic criteria for radiation-associated angiosarcoma typically require a latency period of over three years, both the aforementioned cases and ours involved prior radiation exposure.

**Table 1:** reported cases of paraganglioma with differentiation (including patient demographics, tumor types, anatomical location, S100 immunohistochemical staining results, past medical history, and assessment according to the RAAS criteria)

Study	Age/gender	Composition type	Location	S100	Past medical history	RAAS criteria
Lekovic GP *et al*.	28/female	Paraganglioma-MPNST (radiation-induced sarcoma)	Bilateral carotid artery	Positive	Multiple paraganglioma syndrome	Fulfilled
Razack R *et al*.	44/female	Paraganglioma-MPNST	Cervix	Positive	None	Not fulfilled
Our case	79/female	Paraganglioma-angiosarcoma	Lung	Negative	Carotid paraganglioma	Fulfilled

MPNST: malignant peripheral nerve sheath tumor; RAAS: radiation-associated sarcoma; S100: S100 protein immunostaining

Radiation-induced secondary malignancies (RISMs), including angiosarcoma, are rare but aggressive vascular tumors. However, the term ’radiation-associated angiosarcoma’ (RAAS) is preferred over ’radiation-induced angiosarcoma’ (RIAS), as the latter suggests that radiation is the only causative factor, which may potentially overlook other unknown or poorly understood contributing mechanisms. The diagnostic criteria for radiation-associated sarcoma were first proposed by Cahan *et al*. and later refined by Arlen *et al*. who emphasized that not only the irradiated field but also adjacent tissues are at risk, and established a latency period of at least 3 to 4 years [5,6]. These malignancies occur in only 0.03% to 0.2% of patients within a 10-year post-irradiation period [7]. Our case meets Cahan´s criteria, with a latency period of four years between radiation therapy and the onset of angiosarcoma transformation, supporting the diagnosis of RAAS, but a de novo transformation from paraganglioma to angiosarcoma cannot be totally ruled out.

With advancements in technology, the detection of paragangliomas has significantly improved, particularly through the use of somatostatin receptor scintigraphy and somatostatin analogues. More recently, Ga-68 DOTATATE PET/CT has emerged as the preferred imaging modality due to its high sensitivity and specificity for identifying neuroendocrine tumors. Ga-68 DOTATATE is a radiotracer that targets somatostatin receptors, which are commonly overexpressed in paragangliomas, enabling precise localization of both primary and metastatic lesions. This advanced whole-body imaging technique is especially valuable not only for staging, monitoring treatment response, and detecting recurrence, but also for facilitating early diagnosis. A study by Janssen *et al*. demonstrated that Ga-68 DOTATATE PET/CT achieved a lesion-based detection rate of 97.6%, significantly outperforming other imaging modalities, including 18F-FDG PET/CT, 18F-FDOPA PET/CT, 18F-FDA PET/CT, and conventional CT/MRI [8].

In addition to diagnostic improvements, somatostatin analogues such as Lutetium-177 DOTATATE have shown promising therapeutic potential in paragangliomas, achieving partial response or disease stability in up to 80% of patients with neuroendocrine tumors [9]. Although its use in malignant paragangliomas has been reported in a limited number of cases, severe adverse reactions can occur during or after treatment. These risks may be reduced by modifying the treatment protocol, such as by prolonging the infusion time or lowering the initial dose. Unfortunately, our patient passed away before the treatment could be initiated.

## Conclusion

Our case represents an exceptionally rare presentation of malignant paraganglioma with pulmonary metastasis, further distinguished by the presence of angiosarcomatous differentiation, which is an extraordinarily uncommon histopathological combination not previously documented in the literature. Given the patient´s history of carotid body paraganglioma and prior radiation therapy, the possibility of radiation-associated malignant transformation should also be considered. This case underscores the importance of heightened clinical vigilance for rare tumor variants and the utility of comprehensive immunohistochemical profiling in complex neuroendocrine neoplasms, and emphasizes the need to consider secondary malignancies in paragangliomas with atypical features as a differential diagnosis, which may be associated with a poorer prognosis.
